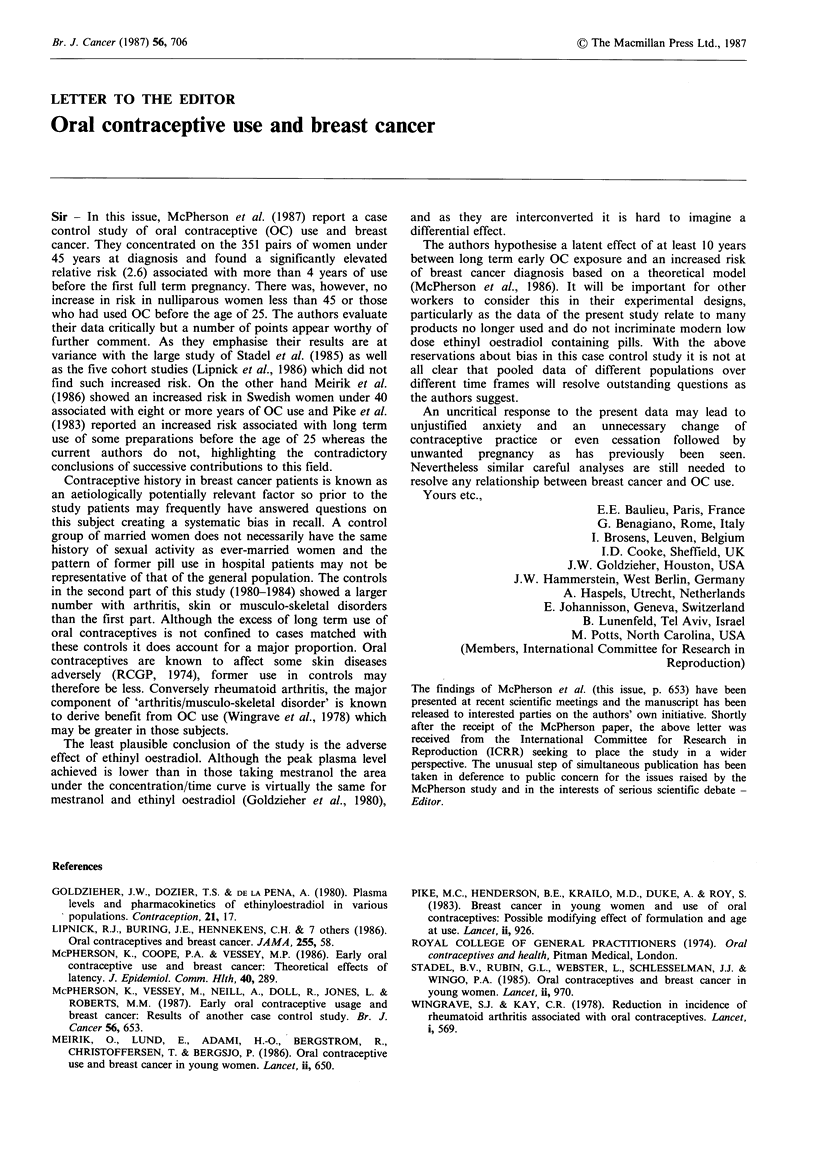# Oral contraceptive use and breast cancer.

**DOI:** 10.1038/bjc.1987.272

**Published:** 1987-11

**Authors:** E. E. Baulieu, G. Benagiano, I. Brosens, I. D. Cooke, J. W. Goldzieher, J. W. Hammerstein, A. Haspels, E. Johannisson, B. Lunenfeld, M. Potts


Br. J. Cancer (1987) 56, 706 © The Macmillan Press Ltd., 1987

LETTER TO THE EDITOR

### Oral contraceptive use and breast cancer

**Sir** – In this issue, McPherson *et al.* (1987) report a case control study of oral contraceptive (OC) use and breast cancer. They concentrated on the 351 pairs of women under 45 years at diagnosis and found a significantly elevated relative risk (2.6) associated with more than 4 years of use before the first full term pregnancy. There was, however, no increase in risk in nulliparous women less than 45 or those who had used OC before the age of 25. The authors evaluate their data critically but a number of points appear worthy of further comment. As they emphasise their results are at variance with the large study of Stadel *et al.* (1985) as well as the five cohort studies (Lipnick *et al.*, 1986) which did not find such increased risk. On the other hand Meirik *et al.* (1986) showed an increased risk in Swedish women under 40 associated with eight or more years of OC use and Pike *et al.* (1983) reported an increased risk associated with long term use of some preparations before the age of 25 whereas the current authors do not, highlighting the contradictory conclusions of successive contributions to this field.

Contraceptive history in breast cancer patients is known as an aetiologically potentially relevant factor so prior to the study patients may frequently have answered questions on this subject creating a systematic bias in recall. A control group of married women does not necessarily have the same history of sexual activity as ever-married women and the pattern of former pill use in hospital patients may not be representative of that of the general population. The controls in the second part of this study (1980–1984) showed a larger number with arthritis, skin or musculo-skeletal disorders than the first part. Although the excess of long term use of oral contraceptives is not confined to cases matched with these controls it does account for a major proportion. Oral contraceptives are known to affect some skin diseases adversely (RCGP, 1974), former use in controls may therefore be less. Conversely rheumatoid arthritis, the major component of 'arthritis/musculo-skeletal disorder' is known to derive benefit from OC use (Wingrave *et al.*, 1978) which may be greater in those subjects.

The least plausible conclusion of the study is the adverse effect of ethinyl oestradiol. Although the peak plasma level achieved is lower than in those taking mestranol the area under the concentration/time curve is virtually the same for mestranol and ethinyl oestradiol (Goldzieher *et al.*, 1980), and as they are interconverted it is hard to imagine a differential effect.

The authors hypothesise a latent effect of at least 10 years between long term early OC exposure and an increased risk of breast cancer diagnosis based on a theoretical model (McPherson *et al.*, 1986). It will be important for other workers to consider this in their experimental designs, particularly as the data of the present study relate to many products no longer used and do not incriminate modern low dose ethinyl oestradiol containing pills. With the above reservations about bias in this case control study it is not at all clear that pooled data of different populations over different time frames will resolve outstanding questions as the authors suggest.

An uncritical response to the present data may lead to unjustified anxiety and an unnecessary change of contraceptive practice or even cessation followed by unwanted pregnancy as has previously been seen. Nevertheless similar careful analyses are still needed to resolve any relationship between breast cancer and OC use.

Yours etc.,

E.E. Baulieu, Paris, France
G. Benagiano, Rome, Italy
I. Brosens, Leuven, Belgium
I.D. Cooke, Sheffield, UK
J.W. Goldzieher, Houston, USA
J.W. Hammerstein, West Berlin, Germany
A. Haspels, Utrecht, Netherlands
E. Johannisson, Geneva, Switzerland
B. Lunenfeld, Tel Aviv, Israel
M. Potts, North Carolina, USA
(Members, International Committee for Research in Reproduction)

The findings of McPherson *et al.* (this issue, p. 653) have been presented at recent scientific meetings and the manuscript has been released to interested parties on the authors' own initiative. Shortly after the receipt of the McPherson paper, the above letter was received from the International Committee for Research in Reproduction (ICRR) seeking to place the study in a wider perspective. The unusual step of simultaneous publication has been taken in deference to public concern for the issues raised by the McPherson study and in the interests of serious scientific debate – *Editor*.

**References**

GOLDZIEHER, J.W., DOZIER, T.S. & DE LA PENA, A. (1980). Plasma levels and pharmacokinetics of ethinyloestradiol in various populations. *Contraception*, **21**, 17.

LIPNICK, R.J., BURING, J.E., HENNEKENS, C.H. & 7 others (1986). Oral contraceptives and breast cancer. *JAMA*, **255**, 58.

McPHERSON, K., COOPE, P.A. & VESSEY, M.P. (1986). Early oral contraceptive use and breast cancer: Theoretical effects of latency. *J. Epidemiol. Comm. Hlth*, **40**, 289.

McPHERSON, K., VESSEY, M., NEILL, A., DOLL, R., JONES, L. & ROBERTS, M.M. (1987). Early oral contraceptive usage and breast cancer: Results of another case control study. *Br. J. Cancer* **56**, 653.

MEIRIK, O., LUND, E., ADAMI, H.-O., BERGSTROM, R., CHRISTOFFERSEN, T. & BERGSJO, P. (1986). Oral contraceptive use and breast cancer in young women. *Lancet*, **ii**, 650.

PIKE, M.C., HENDERSON, B.E., KRAILO, M.D., DUKE, A. & ROY, S. (1983). Breast cancer in young women and use of oral contraceptives: Possible modifying effect of formulation and age at use. *Lancet*, **ii**, 926.

ROYAL COLLEGE OF GENERAL PRACTITIONERS (1974). *Oral contraceptives and health*, Pitman Medical, London.

STADEL, B.V., RUBIN, G.L., WEBSTER, L., SCHLESSELMAN, J.J. & WINGO, P.A. (1985). Oral contraceptives and breast cancer in young women. *Lancet*, **ii**, 970.

WINGRAVE, S.J. & KAY, C.R. (1978). Reduction in incidence of rheumatoid arthritis associated with oral contraceptives. *Lancet*, **i**, 569.